# Can Building “Artificially Intelligent Cities” Safeguard Humanity from Natural Disasters, Pandemics, and Other Catastrophes? An Urban Scholar’s Perspective

**DOI:** 10.3390/s20102988

**Published:** 2020-05-25

**Authors:** Tan Yigitcanlar, Luke Butler, Emily Windle, Kevin C. Desouza, Rashid Mehmood, Juan M. Corchado

**Affiliations:** 1 School of Built Environment, Queensland University of Technology, 2 George Street, Brisbane, QLD 4000, Australia; luke.butler@hdr.qut.edu.au (L.B.); emily.windle@connect.qut.edu.au (E.W.); 2 QUT Business School, Queensland University of Technology, 2 George Street, Brisbane, QLD 4000, Australia; kevin.desouza@qut.edu.au; 3 High Performance Computing Center, King Abdulaziz University, Al Ehtifalat St, Jeddah 21589, Saudi Arabia; rmehmood@kau.edu.sa; 4 Bisite Research Group, University of Salamanca, 37007 Salamanca, Spain; corchado@usal.es; 5 Air Institute, IoT Digital Innovation Hub, 37188 Salamanca, Spain; 6 Department of Electronics, Information and Communication, Faculty of Engineering, Osaka Institute of Technology, Osaka 535-8585, Japan; 7 Pusat Komputeran dan Informatik, Universiti Malaysia Kelantan, Kelantan 16100, Malaysia

**Keywords:** artificial intelligence (AI), artificially intelligent city, artificially intelligence commons, smart city, smart urban technology, urban informatics, sustainable urban development, climate change, pandemics, natural disasters

## Abstract

In recent years, artificial intelligence (AI) has started to manifest itself at an unprecedented pace. With highly sophisticated capabilities, AI has the potential to dramatically change our cities and societies. Despite its growing importance, the urban and social implications of AI are still an understudied area. In order to contribute to the ongoing efforts to address this research gap, this paper introduces the notion of an artificially intelligent city as the potential successor of the popular smart city brand—where the smartness of a city has come to be strongly associated with the use of viable technological solutions, including AI. The study explores whether building artificially intelligent cities can safeguard humanity from natural disasters, pandemics, and other catastrophes. All of the statements in this viewpoint are based on a thorough review of the current status of AI literature, research, developments, trends, and applications. This paper generates insights and identifies prospective research questions by charting the evolution of AI and the potential impacts of the systematic adoption of AI in cities and societies. The generated insights inform urban policymakers, managers, and planners on how to ensure the correct uptake of AI in our cities, and the identified critical questions offer scholars directions for prospective research and development.

## 1. Introduction

### What Is an Artificially Intelligent City?

During the current Anthropocene era—the geological epoch which has had significant human impact on Earth’s geology and ecosystems—we have developed technological capabilities that have enabled us to greedily use limited natural resources for economic profit [1,2]. This ruthless capitalist practice not only brought about anthropogenic climate change, but also caused socioeconomic inequalities to soar globally [3].

In recent years, technology, as part of knowledge-based development efforts [4], has been viewed as the solution to severe environmental, economic, and social crises [5,6]. Consequently, the smart city concept has come to the forefront of discourses on urban planning and development [7]. Accordingly, many see emerging technologies, particularly artificial intelligence (AI), as a way to safeguard our civilization from the catastrophic consequences of climate change [8], biodiversity loss [9], natural disasters [10], unsustainable development [11], pandemics [12], and so on.

**Figure 1:**
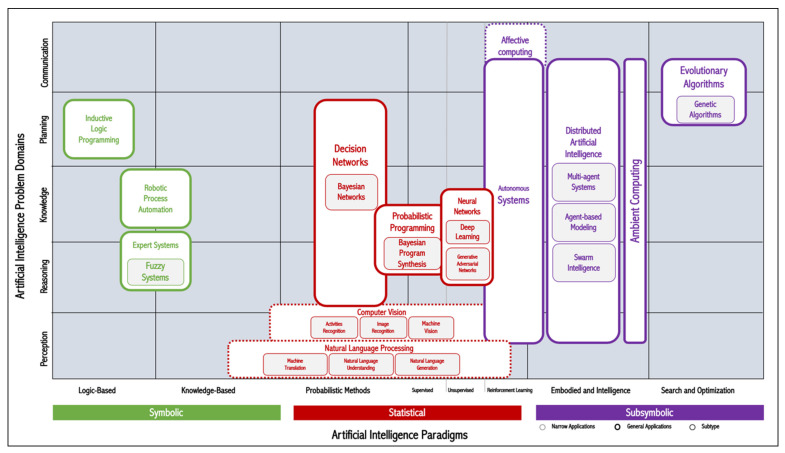
Classification of AI-driven computational techniques, derived from Corea [14].

Simply, AI is defined as “machines or computers that mimic cognitive functions that humans associate with the human mind, such as learning and problem solving” [13]. AI-driven computational techniques are diverse and range from rule-based systems to deep learning systems. A popular AI knowledge map was created by Corea [14]. His conceptualization brings together the AI paradigms and problem domains (Figure 1).

The AI paradigm and its technology-enabled solutions—whether it is autonomous driving, home automation (so-called domotics), robotics, chatbots, or advanced data analytic tools—have opened up new opportunities for cities, where most of the world population resides, where most of the production and consumption activities take place, and also where most of the negative environmental externalities are generated [15,16]. While some scholars see AI as an opportunity to advance smart cities (or smartness of cities) [17,18,19,20,21], others see AI generating a whole new city brand, especially when the AI applications become mainstream in our cities [22]. In other words, in the near future, we will see a trend to build ‘artificially intelligent cities’ from scratch, or to retrofit traditional cities, converting them into artificially intelligent ones.

We define an artificially intelligent city as an urban locality functioning as a robust system of systems, and whose economic, societal, environmental, and governmental activities are based on sustainable practices driven by AI technologies, helping us achieve social good and other desired outcomes and futures for all humans and non-humans.

In the age of smart cities—where urban locations are starting to be wired with smart technologies including sensor networks—and given the highly sophisticated capabilities of AI, we foresee a potential dramatic change in our cities and societies [23]. There is, hence, an increasing need to investigate the urban and social implications of AI. This is an understudied area of research, and a gap in the literature on AI and city/society.

We also note that there are different levels of AI, including: (a) reactive machines (e.g., IBM’s Deep Blue); (b) limited memory AI (e.g., chatbots, virtual assistants, self-driving vehicles); (c) theory of mind AI (a concept that is in progress at the moment); and (d) self-aware AI (only hypothetical at this stage) [24]. There is also another categorization of levels of AI, such as: (a) artificial narrow intelligence (represents all of the existing AI today); (b) artificial general intelligence (its main idea is that AI agents can learn, perceive, understand, and function completely like a human being); and (c) artificial superintelligence (an idea that AI replicates the multifaceted intelligence of human beings and becomes exceedingly better at everything it does) [25]. The disruption of each level of AI will be different in our cities and societies. Throughout this paper, we focus on the current level of AI: artificial narrow intelligence.

Against this backdrop, we prepared this viewpoint in order to help in bridging this gap along with promoting further research on the topic. In this paper, we introduce a provocative artificially intelligent city notion as the potential successor of the currently popular smart city concept, where city smartness today is increasingly depending on the use of viable technology solutions, including AI. The paper, by placing the AI literature, developments, trends, and applications under the microscope, provides a commentary on whether building artificially intelligent cities can safeguard humanity from natural disasters, pandemics, and other catastrophes.

## 2. Conceptual and Practical Background

### 2.1. Has the Artificial Intelligence Era Already Begun?

**Figure 2:**
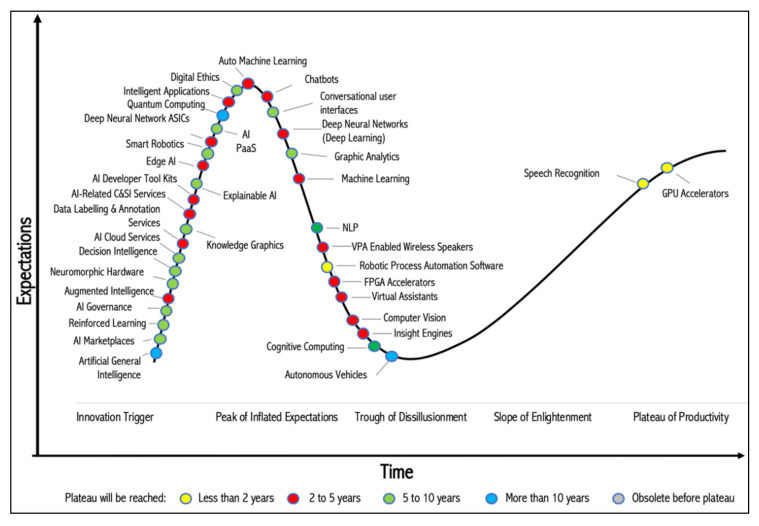
Hype cycle of AI applications, derived from Gartner [28].

AI is one of the most disruptive technologies of our time and its capabilities have progressed rapidly [26]. The uptake of AI in organizations is on the rise. For instance, between 2018 and 2019, the number of organizations that deployed AI grew from 4% to 14%, and among the AI applications, conversational AI is at the top of corporate agendas spurred by the worldwide success of Amazon Alexa, Google Assistant, and Apple’s Siri [27]. Gartner [28] provides insights into the hype cycle for AI applications, which reflects the growing popularity of machine learning, intelligent applications, and AI-as-a-Service (AIaaS) or AI-Platform-as-a-Service (AI-PaaS) (Figure 2).

**Figure 3:**
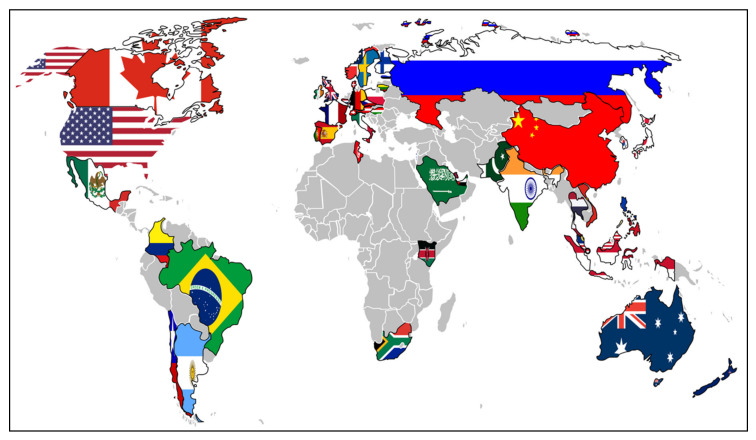
Countries with a national AI strategy, derived from Holon IQ [29].

**Figure A1:**
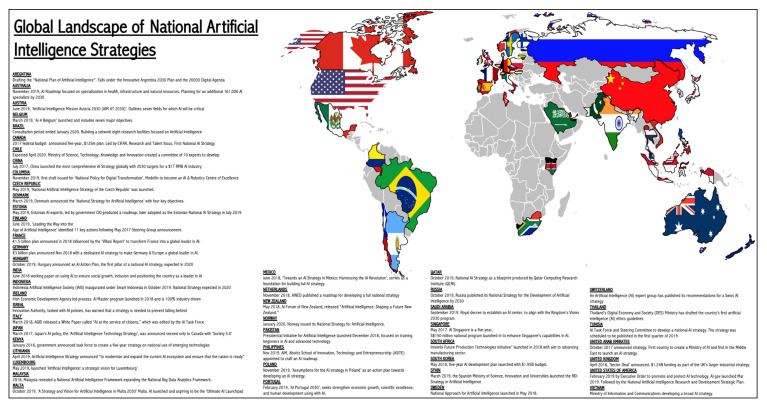
Global landscape of national artificial intelligence strategies, derived from Holon IQ [29].

In recent years, governments around the world have started to see AI as a nation-defining and global economic competitiveness-increasing capability [29]. In recognition of the increasing importance of AI, as of February 2020, 50 countries have already developed specific national AI strategies—where these countries represent 90% of global gross domestic product (GDP). Figure 3 illustrates the location of these countries; a brief further info on each country’s national strategy is provided in Appendix A (Figure A1).

AI-driven computational techniques are diverse and wide-ranging. For example, AI has been in use for quite some time in the tasks that are risky or cause harm to humans. This includes the use of automated robots for bomb detection or combat of unmanned aerial vehicles, and the use of autonomous trucks in the mining industry or mobile reconnaissance units for space exploration [30].

AI-enabled applications include robotic processes [31] for automating public sector tasks, and autonomous delivery bots [32] and chatbots [33] for enhancing business intelligence, stakeholder engagement experience, and customer service quality. Today, AI is rapidly changing the nature of jobs. Many of the services that have been offered by human workers are now being revolutionized by technology. For example, chatbots automate the work of information technology (IT) professionals [34] and human resource (HR) departments [35], so that they can focus on higher value tasks.

Autonomous vehicles and driverless shuttle buses are being trialed worldwide. Driverless shuttle bus services are expected to start carrying fare-paying customers in Scotland later in 2020 [36]. Likewise, robot police services are planned to be launched in Houston, Texas to curb petty crime and free up law enforcement resources in 2020 [37].

AI-based systems are providing various solutions. These solutions facilitate the creation of new products and services in many different fields. Particularly, sensor networks are undergoing great expansion and development and the combination of both AI and sensor networks has now become a reality to change our lives and our cities. The integration of these two prominent technologies—including AIoT (AI-of-Things)—also benefits other areas such as Industry 4.0, Internet-of-Things (IoT), demotic systems, and so on [38,39].

AI is being employed to model the spread of COVID-19 to assist decision makers in understanding the future implications of the virus and the measures that should be taken to limit its spread [40]. For instance, in China, AI is being used to minimize the spread of COVID-19 by mobilizing robots that do cleaning and food preparation tasks [41]. Moreover, the European Union [42] launched the EU vs. Virus challenge via a Pan-European hackathon to find ways to tackle COVID-19 via AI and other applications.

AI also has the potential to help in addressing some of the planetary challenges (Table 1). The World Economic Forum [43] underlines the following eight AI applications as “game changers”: (a) autonomous and connected electric vehicles; (b) distributed energy grids; (c) smart agriculture and food systems; (d) next-generation weather and climate prediction; (e) smart disaster response; (f) AI-designed intelligent, connected, and livable cities; (g) a transparent digital earth; and (h) reinforcement learning for earth sciences breakthroughs.

### 2.2. How Is Artificial Intelligence Being Utilized in Cities?

In the previous section, we have provided some examples of the use of AI in cities. Here in this section, we share a few more examples to cover some of the other aspects of AI for cities. In particular, the AI solutions implemented in Australia have been taken as an example. Like many other advanced knowledge and innovation economies, AI is a rapidly growing field in Australia. Furthermore, the country has been an early adopter of smart technologies [44], particularly for targeting industrial and urban sustainability outcomes [45,46,47]. Some of the existing AI applications and experienced challenges in the country are discussed as follows:

▪ Autonomous vehicles and driverless shuttle buses are being trialed throughout Australia, in all capital cities and some regional centers [48]. Nevertheless, the regulation efforts of autonomous vehicles are yet to follow the autonomous driving trials and developments.
▪ State of NSW police have been using AI systems to identify drivers illegally using mobile phones [49]. These systems review images, detect offences, and then exclude non-offenders. Nonetheless, images are then authorized following a review by human operators.
▪ The importance of review of AI outputs by human operators was highlighted by the Australian federal government’s incorrect use of AI for automatic detection of Centrelink debt and issuing of infringement notices without human input [50]. The process resulted in some individuals receiving notices incorrectly and placed the onus of proof onto the accused.
▪ Other issues have resulted from facial recognition software used in surveillance and crime prevention, which may have unfairly discriminated against Aboriginal and Torres Strait Islanders [51].

**Figure 4:**
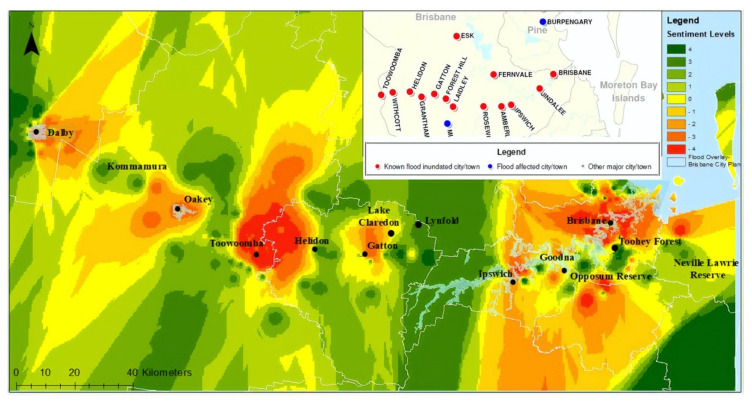
AI and big data analytics in natural disaster management, derived from Kankanamge et al. [59].

Despite these issues, development of AI continues in a variety of fields in Australia, and has been investigated for its use in product/goods delivery [52], environmental and transport monitoring [53], disaster prediction [54], healthcare [55], infrastructure [56], data privacy [57], and agriculture [58]. Just to provide some examples, AI’s contributions to healthcare practice are listed in Table 2. Additionally, AI applications have been used in big data analytics, such as its use in social media analytics to aid natural disaster management. Figure 4 is an example of the disaster severity map generated for the 2010–2011 Queensland Floods with the help of machine learning technology [59].

In terms of strategizing AI, there have been some promising developments in Australia. The most notable one is the AI roadmap, codeveloped by CSIRO’s Data61 and the Australian Government Department of Industry, Innovation and Science. The roadmap identifies strategies to help develop a national AI capability to boost the productivity of Australian industry, create jobs and economic growth, and improve the quality of life for current and future generations. The roadmap emphasizes the need to concentrate on the three key domains: (a) natural resources and the environment; (b) health, aging, and disability; and (c) cities, towns, and infrastructure [61]. Table 3 below elaborates the objectives of these AI domains. Additionally, OECD’s [62] AI policy observatory provides a useful repository of AI in Australia.

## 3. Discussion

### 3.1. Can Artificial Intelligence Help Cities Become Smarter?

Cities are complex organisms and their complexity increases exponentially as they continue to grow [63]. With computational abilities vastly superior to humans, when it comes to ingesting large swaths of data, AI systems are among the core elements of most smart city projects [64].

Other smart technologies such as internet-of-things (IoT) [65], autonomous vehicles (AV) [66,67], big data [68], 5G wireless communication [69], robotics [70], blockchain [71], cloud computing [72], 3D printing [73], virtual reality (VR) [74], augmented reality (AR) [75], digital twins [76], and so on are also transforming our cities [77].

For instance, it is increasingly common to combine machine learning with other emerging technologies to generate advanced urban solutions. Examples include: the use of deep learning and high-performance computing (HPC) for traffic predictions using sensor data [78], incident prediction [79], disaster management [80], and rapid transit systems designed to optimize urban mobility systems [81]. Machine learning has also been used with big data technologies and social media for logistics and urban planning [82,83], event detection for urban governance [84], disease detection [85], and identifying the sources of noise pollution at the city scale [86].

**Figure 5:**
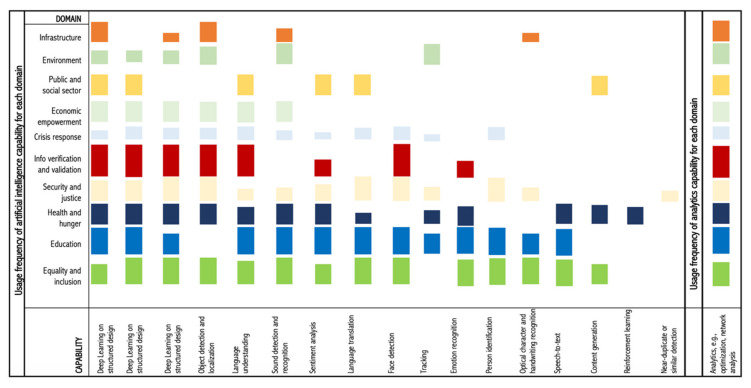
AI capabilities and their use by domains, derived from McKinsey Global Research Institute [93].

Additionally, machine learning has been applied along with distributed computing to improve basic scientific computing operations that are fundamental to urban design modeling methodologies [87]. Moreover, machine learning is paired with IoT for human activity recognition [88], smart farming [89], and developing next-generation distance learning systems [90]. Furthermore, machine learning benefits from data fusion in ubiquitous IoT environments [91], where this creates a potential to significantly enhance AV decision capabilities [92]. Figure 5 lists AI capabilities and their use by domains.

Nevertheless, it is when AI is combined with these technologies that we can really see its big potential to address complex challenges and harness opportunities within our urban environments—given that some ethical issues are adequately addressed. Despite the AI and ethics issue being discussed in academic and government circles, so far only limited guiding principles have been produced and legislated [94]. In that regard, the European Parliament’s [95] initiative on guidelines for the European Union (EU) on ethics in AI is a commendable but limited attempt, as ethical rules on AI are so far essentially of a self-regulatory nature, and there is growing demand for more government oversight.

### 3.2. What Are the Promises and Pitfalls of Artificial Intelligence for Cities?

**Figure 6:**
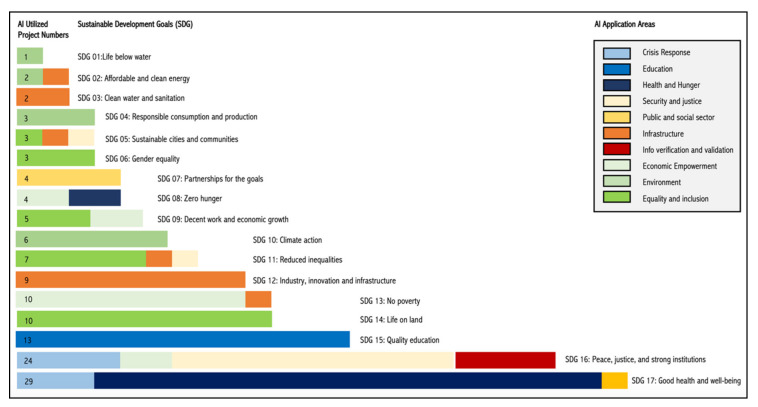
AI utilization for achieving sustainable development goals, derived from McKinsey Global Research Institute [93].

A recent study [96] that evaluated the levels of smartness of Australian local government areas advocated for the importance of integrating urban technologies, including AI, into local service delivery and governance, for instance, the use of AI in tasks that enhance environmental sustainability, such as sorting waste for recycling [97]. Additionally, the practice review conducted by McKinsey Global Research Institute [93] discloses projects from across the globe where AI is utilized for achieving UN’s sustainable development goals (SDG) (Figure 6). Nevertheless, before AI is implemented on a wider scale, it is important to understand how this technology can contribute to making our cities (and the planet) smarter. Conversely, understanding the pitfalls of AI will enable us to ensure AI delivers the desired outcomes in urban areas and beyond.

With the above-mentioned issue in mind, our team in another study [22] evaluated the promises and pitfalls of AI for cities according to the main smart city dimensions of economy, society, environment, and governance [98]. Table 4 below summarizes the key findings of the study.

### 3.3. What Are the Ways to Maximize Artificial Intelligence Promises and Minimize Pitfalls?

The biggest pitfalls of AI-enabled solutions are that they may aggravate the existing socioeconomic disparity [99] and have privacy [100] (for example, increased government surveillance during COVID-19) and cybersecurity [101] issues. Most of our cities are already fragile and inattention to how local governments maintain social compacts will only increase their fragility [102]. It is imperative that technological progress does not accelerate the widening of existing fractures, or incubate new sources of fractures, in our cities [103].

Given the fast-paced implementation of AI, it is important that we act now and find ways to minimize the pitfalls of AI while maximizing its promises [104]. Some of the useful actions are presented below.

▪ The first step should be to engage multiple stakeholders [105]. Active collaboration among people from a wide range of industries and backgrounds can help highlight the promises of AI technology, identify pitfalls, and improve trust. This will also contribute to humanizing AI.
▪ Secondly, paramount to developing trust is demonstrating the ability of AI technology to ensure data security and reduce vulnerabilities [106], including hacking and misinformation.
▪ Thirdly, AI technology should be agile, so that it can cope with uncertainty [107]. It must also be frugal so it can be implemented in a way that does not lead to wasting public resources on failed attempts and does not become obsolete.
▪ Additionally, regulation is crucial for controlled implementation [108]—standards and ethical frameworks help ensure AI is deployed responsibly and in keeping with public values.
▪ Furthermore, more research and development (R&D) is required to ensure the cascading effects of AI, across the various levels of a city (local, neighborhood, city, and the larger regional ecosystem) and society. Deploying AI systems calls for an assessment of their impact on a system of systems [109].
▪ Next, it is critical to develop AI solutions with a public research consortium to ensure that technology is not solely used as a means of gaining profit.
▪ Finally, it is also important to consider the intended, as well as the unintended, consequences of AI [110] that will arise not only within one system (e.g., economic) but across the collection of interrelated systems (e.g., interaction between economic, social, and physical infrastructures).

## 4. Conclusions

### 4.1. Are Artificially Intelligent Cities on the Horizon?

According to Andrew Ng, cofounder of Google Brain, “AI is the new electricity. Just as 100 years ago electricity transformed industry after industry, AI will now do the same.” The impact of AI will go beyond the industry; it is set to change the world [111]. An internationally conducted survey [112] highlighted that “the prospect of an AI future both excites and concerns people around the globe. Nonetheless, fears around the drawbacks of AI are offset by the benefits, and the net result is positive. AI will likely to change society for the better.” AI applications have also significant potential to transform our cities. This may lead to the next-generation smart cities [113] being coined as “artificially intelligent cities”. Building artificially intelligent cities may save our civilization from the earlier mentioned catastrophes, but it all depends on how we design and use AI, and on who will profit from it [114]. The risk here is for AI to become a vehicle for increasing the wealth of the top 1% of income earners (i.e., top 10 wealthiest people in the world and monopolistic multinational corporations) and the power of biased and unethical politicians [115].

Time will tell if AI systems make our cities “smart enough” to provide better living conditions for all (i.e., people, flora, and fauna coexisting in urban ecosystems). To date, while there have been significant technological advances, these have not been matched with innovations in governance mechanisms. In addition, the policy apparatuses of most local governments need significant modernization to take full advantage of technology affordances in an agile manner [116].

If there is one thing that cities and local governments have learned from the ongoing COVID-19 pandemic, caused by the SARS-CoV-2 virus, is that when they are willing, they can respond in a proactive and agile manner to the changing environmental conditions. We hope that cities will keep on this track after the current crises pass, modernizing their governance mechanisms and policy frameworks to take full advantage of emerging technologies—particularly AI.

The COVID-19 pandemic has also demonstrated that local governments need to seriously consider their digital infrastructure capabilities and capacities. For example, when the Queensland state government (in Australia) decided to make education available online, its infrastructure failed to deliver the public service (i.e., provision of online education) due to significant web traffic. A few schools had backups in place with paper resources but the problem highlighted significant issues with existing networks [117]. Online education has also highlighted social equity issues associated with the digital divide with some lower income students struggling to meet the required technology capabilities [118], and special needs students, including those who speak a language other than English, struggling to receive the required one-on-one assistance [119].

In this paper, we mainly focused on the artificial narrow intelligence level of AI. Nevertheless, if somehow one day we manage to build artificial general intelligence or artificial superintelligence (these two AI levels also correspond to singularity, that is, in simple terms, the intelligence explosion), we need to do all it takes for it to be, as Tegmark [120] calls it, a “Friendly-AI” (a superintelligence whose goals are aligned with ours). Speculation on how to build artificial general intelligence or artificial superintelligence or singularity that would reshape our cities, societies, and civilization is beyond the scope of this viewpoint.

### 4.2. What Are the Key Lines of Research Concerning Artificial Intelligence and Cities?

There are some important issues, in the context of AI and cities, that prospective research must address in order to provide our cities and societies with the best technological outcomes. We strongly believe that further investigating some of the critical issues in prospective research projects by scholars of this highly interdisciplinary field will shed light on the better conceptualization and practice of AI (artificial narrow intelligence level) in the context of cities and societies. These issues are listed below:

▪ How can AI systems be developed for cities that are robust, less hackable, and are not used to manipulate and control populations (e.g., voting for a politician/political party)?
▪ How can we best tackle the AI pitfalls to assure positive outcomes for cities and societies (e.g., security, privacy, regulations, and inequality)?
▪ How can we avoid heavy reliance on automated decision-making systems, making the society passive or inactive in determining its goals?
▪ How can AI-induced decisions or solutions in cities be more participatory, democratic, and transparent?
▪ How can AI be utilized best in cities to achieve desired urban outcomes for all (i.e., human and non-human)?
▪ How can we determine the best possible scenarios and factors of success and failure in implementing AI in cities?
▪ How can we determine the best approach to start building artificially intelligent cities (e.g., from scratch, retrofitting, or a combination of both)?
▪ How can the uniqueness, image, or character of each city and society be maintained given AI is in the play and there might be one best solution?
▪ How can we design AI systems for cities that preserve, and even promote, societal values and cultural heritage and historic artifacts (e.g., embrace legacy), while simultaneously exploiting emerging technologies and contemporary platforms?
▪ How can we form the AI commons and ensure that AI can achieve its potential for social good?
▪ How can local governments meet the need for rich, real-time, location- and context-specific data and preserve privacy and security, while designing AI systems?
▪ How can the negative environmental externalities of large AI technology and systems be minimized?
▪ How can the blueprints be developed for the next global transformation of cities to create carbon-free and adaptive futures for humanity?

Considering AI’s current ability to ingest big data for exploratory studies and real-time decision-making, it would be worthwhile to address the following research question (in addition to the above list): How can AI be used to find what we may have missed in terms of developing better (e.g., fairer and more productive) social structures, social geography, social good, political structures, economic structures, energy sources, modes of transportation, design of living structures and spaces (i.e., in normal and disaster times, such as those that COVID-19 and similar pandemics could bring on us), and so on?

The concept of AI advising us on human sociology and similar matters may sound very offensive to some, but when properly done, AI is merely a tool that can be used by humans for their advantage (in the sense of artificial narrow intelligence). Humans tend to learn and incrementally apply the acquired knowledge into practice. AI can analyze ideas for us faster and more in depth, and together with other developments in technologies (e.g., AR, high-performance computing, IoT, and big data), it could allow us to study and predict the potential harms and benefits of alternative ideologies, and develop better futures. Moreover, AI, in the context of artificially intelligent cities, can also help us transform our cities into smarter and more prosperous and creative ones [121,122,123].

Lastly, we conclude the paper by elaborating on the question we raised in the title of this paper—Can building artificially intelligent cities safeguard humanity from natural disasters, pandemics, and other catastrophes? The existing AI literature reviewed in this paper, unfortunately, does not allow us to answer it with a confident “yes”. The answer to this question depends on the findings of the studies focusing on the above-listed critical questions. While we continue to have hope that AI technology will help fix or at least ease the problems created by us, perhaps another important issue is whether we will be able to use AI for the common good of all—rather than the so-called 1% [124] that is already in control of the world economy.

On that very point, at his Turing Lecture on deep learning for AI, Yoshua Bengio [125] highlighted the critical importance of using AI for social good and introduced two actionable items: (a) favoring machine learning applications to help the poorest countries fight climate change, improve healthcare and education, and so on; and (b) forming the concept of AI commons and coordinate, prioritize, and channel funding for the use of AI for social good. As stated by Hager et al. [126], “AI can be a major force for social good; but it depends on how we shape this new technology, and the questions we use to inspire young researchers.”

## Figures and Tables

**Table 1:** AI application areas for addressing planetary challenges, derived from World Economic Forum [43].

Planetary Challenges	AI Application Areas
Climate change	Clean power
	Smart transport options
	Sustainable production and consumption
	Sustainable land use
	Smart cities and homes
Healthy oceans	Fishing sustainability
	Preventing pollution
	Protecting habitats
	Protecting species
	Impacts from climate change (including acidification)
Clean air	Filtering and capture
	Monitoring and prevention
	Early warning
	Clean fuels
	Real-time, integrated, adaptive urban management
Biodiversity and conversation	Habitat protection and restoration
	Sustainable trade
	Pollution control
	Invasive species and disease control
	Realizing natural capital
Water security	Water supply
	Catchment control
	Water efficiency
	Adequate sanitation
	Drought planning
Weather and disaster resilience	Prediction and forecasting
	Early warning systems
	Resilient infrastructure
	Financial instruments
	Resilience planning

**Table 2:** AI applications and motivation for adoption in healthcare practice, derived from Park [60].

Application	Motivation for Adoption
Robot-assisted surgery	Technological advances in robotic solutions for more types of surgery
Virtual nursing assistants	Increasing pressure caused by medical labor shortage
Administrative workflow	Easier integration with existing technology infrastructure
Fraud detection	Need to address complex service and payment fraud attempts
Dosage error reduction	Prevalence of medical errors, which leads to tangible penalties
Connected machines	Proliferation of connected machines and devices
Clinical trial participation	Client cliff, plethora of data, outcomes-driven approach
Preliminary diagnosis	Interoperability and data architecture to enhance accuracy
Automated image diagnosis	Storage capacity, greater trust in AI technology
Cybersecurity	Increase in breaches, pressure to protect health data

**Table 3:** Priority AI specialization domains and their objectives, derived from Data61 [61].

Domain	Objective
Natural resources and the environment	Developing AI solutions for enhanced natural resource management to reduce the costs and improve the productivity of agriculture, mining, fisheries, forestry, and environmental management
Health, aging, and disability	Developing AI solutions for health, aging, and disability support to reduce costs, improve wellbeing, and make quality care accessible for all Australians
Cities, towns, and infrastructure	Developing AI solutions for better towns, cities, and infrastructure to improve the safety, efficiency, cost-effectiveness, and quality of the built environment

**Table 4:** Promises and pitfalls of AI for cities, derived from Yigitcanlar et al. [22].

Domains	Promises	Pitfalls
Economy	▪ Enhance productivity and innovation ▪ Reduce costs and increase resources ▪ Support the decision-making process ▪ Automate decision-making	▪ Biased decision-making ▪ Unstable job market ▪ Loss of revenue streams ▪ Loss of employment ▪ Economic inequality
Society	▪ Improve healthcare monitoring ▪ Enhance health diagnosis outcomes ▪ More adaptable education system ▪ More personalized teaching ▪ Task optimization	▪ Biased decision-making ▪ Misdiagnosis ▪ Unstable job market ▪ Loss of employment ▪ Data privacy and security
Environment	▪ Assist environmental monitoring ▪ Optimize energy consumption ▪ Optimize energy production ▪ Optimize transport systems ▪ Assist in developing more environmentally efficient transport systems	▪ Biased decision-making ▪ Increased urban sprawl ▪ More kilometers traveled by motor vehicles ▪ Changed property values ▪ Energy intensive technology ▪ Increased carbon footprint
Governance	▪ Enhance surveillance systems ▪ Improve cyber safety ▪ Aid in disaster management planning and operations ▪ Assist citizens with new technologies	▪ Biased decision-making ▪ Racial bias and discrimination ▪ Suppression of public voice/protest ▪ Violation of civil liberties ▪ Privacy concern ▪ Unethical use of technology ▪ Risk of misinformation ▪ Cybersecurity concern
